# Barriers and facilitators of psychosocial service provision for patients with cancer across six hospitals in Ethiopia: a qualitative study

**DOI:** 10.3389/fpsyt.2025.1689641

**Published:** 2025-11-25

**Authors:** Winini Belay, Finina Abebe Ware, Mirgissa Kaba, Adamu Addissie, Eva Johanna Kantelhardt, Abigiya Wondimagegnehu

**Affiliations:** 1 Department of Epidemiology and Biostatistics, School of Public Health, College of Health Sciences, Addis Ababa University, Addis Ababa, Ethiopia; 2 Global Health Working Group, Institute of Medical Epidemiology, Biostatistics and Informatics, Martin-Luther University, Halle (Saale), Germany; 3 Clinton Health Access Initiative (CHAI), Addis Ababa, Ethiopia; 4 Department of Preventive Medicine, School of Public Health, College of Health Sciences, Addis Ababa University, Addis Ababa, Ethiopia; 5 Department of Gynecology, Martin-Luther-University, Halle (Saale), Germany

**Keywords:** cancer, psychosocial support, barriers, facilitators, integration and routine care

## Abstract

**Background:**

While psychosocial services are known to improve treatment adherence and quality of life for cancer patients by mitigating anxiety and depression, evidence from Ethiopia is limited. A recent trial introduced integrated psychosocial interventions, including counseling, group discussions, educational materials, and home visits, into routine care. The present study explores barriers and facilitators affecting psychosocial service provision in selected Ethiopian hospitals.

**Method:**

A qualitative study was conducted at six hospitals across four regions of Ethiopia, where psychosocial support had been introduced and provided to patients with cancer. Data were collected through in-depth interviews (IDIs) and focus group discussions (FGDs) with patients diagnosed with breast, cervical, or colorectal cancer; as well as key informant interviews (KIIs) with healthcare professionals, including oncologists, gynecologists, surgeons, nurses, and health extension workers. All interviews were transcribed, translated and reviewed for completeness. To enhance data familiarity, transcripts and audio recordings were reviewed multiple times. NVivo software was used for data management and organization. Data was coded inductively while predefined themes are introduced deductively, followed by thematic analysis to identify key patterns and insights.

**Result:**

Barriers to psychosocial support (PSS) in cancer care include limited awareness of its importance, as treatment is often considered to be purely medical. Although home visits are common in maternal health, in cancer care, they face resistance due to unfamiliarity. Disclosure challenges also persist, with providers avoiding sensitive conversations, leaving patients under-informed. Hospital leadership tends to prioritize physical care over PSS. However, survivor stories enhance patient reassurance and openness; travel reimbursements and refreshments facilitate patient participation and communication, and routine supervision of PSS activities supports provider effectiveness in PSS provision.

**Conclusion:**

Integrating PSS into routine cancer care requires a shift in the mindset of patients, providers, and leadership, recognizing PSS as an essential component of comprehensive cancer care. Raising awareness about home visits and strengthening provider skills through targeted training on disclosure can improve patient engagement and quality of care.

## Background

Cancer diagnosis and treatment often disrupt psychological and social well-being of patients, contributing to mental health conditions such as anxiety, depression and adjustment disorders (1, 2). These mental health conditions can compromise treatment adherence, diminish quality of life, and worsen clinical outcomes of patients with cancer; hence, addressing the psychosocial dimensions of cancer care is crucial to enhancing patient-centered outcomes (3).

Psychosocial support, encompassing counseling, education, coping strategies, and support groups, is increasingly recognized as a core component of oncology care in high-income countries (4), where it is routinely embedded within multidisciplinary treatment frameworks. However, in low- and middle-income countries (LMICs), including Ethiopia, such integration remains limited (5). Structural constraints, workforce shortages, and low awareness among both providers and patients hinder the availability and uptake of psychosocial services in these settings (6), with the absence of standardized service models and limited institutional support further complicating these challenges (7).

In Ethiopia, oncology services are largely centralized, and psychosocial care is rarely incorporated into routine service in cancer care (8). While isolated initiatives, such as breast cancer support groups, have demonstrated potential to foster emotional resilience and reduce stigma (9), these efforts often lack systematic evaluation. Despite the growing recognition of the importance of psychosocial support, little is known about how healthcare providers in Ethiopia navigate the practical challenges of implementing such services in resource-constrained settings. To address this gap, our previous trial investigated the implementation of psychosocial services using a Social and Behavior Change Communication (SBCC) model, incorporating counseling, informational brochures provision, support group discussions, audiovisual materials, and home visits, delivered across six hospitals to patients with breast, cervical, colorectal, and prostate cancers (10).

The effects of the service provision on anxiety, depression, quality of life, and treatment adherence were evaluated. However, several barriers emerged, hindering the effective provision of these services. Therefore, as a continuation of the prior trial, the present study aims to explore the facilitators and barriers that hinder the provision of psychosocial services in the implementation hospitals. Identification of these factors will enhance the effective integration of these services by offering a context-specific framework for integrating psychosocial services into routine cancer care, contributing actionable insights for policy, training, and service design, ultimately enhancing patient health outcomes and quality of life.

## Materials and methods

### Study setting

This study was conducted at six hospitals that provide cancer treatment services and had participated in the psychosocial service provision initiative, located in four regions of Ethiopia: Southern Ethiopia, Sidama, Central Ethiopia, and Oromia. Two hospitals are located in urban settings: Tikur Anbessa Specialized Hospital (Addis Ababa) and Adama Hospital Medical College (Adama, southeast Ethiopia). The other four hospitals are located in semi-urban areas: Butajira General Hospital (Central Ethiopia), Nigist Eleni Mohammed Memorial Comprehensive Specialized Hospital (Central Ethiopia), St. Luke’s Catholic Hospital and College of Nursing and Midwifery (Southwest), and Assela Teaching and Referral Hospital (South-central Ethiopia).

### Study design

A qualitative exploratory design was employed as a continuation of a larger cluster randomized controlled trial that integrated psychosocial service packages into routine cancer care. This follow-up qualitative study aimed to identify barriers and facilitators influencing psychosocial service provision across the six implementation hospitals involved in the prior trial.

### Psychosocial service intervention

Psychosocial services, including counseling, informational brochures, audiovisual materials (such as survivor stories and educational videos), support group discussions, and periodic home visits, were provided to patients diagnosed with cancer, following comprehensive training for health professionals. Nurses and health extension workers were key implementers: nurses led counseling sessions, organized support group discussions, and distributed informational brochures, while health extension workers conducted home visits. The services were delivered as a package for six months at the selected health facilities, with weekly supervision to support providers.

### Data collection and procedures

This study employed a hybrid inductive–deductive approach, which influenced the design of the interview guides and the overall data collection strategy. Deductive elements were guided by the study’s conceptual framework and existing literature on barriers and facilitators to psychosocial service provision in low-resource settings. These informed the development of core questions aligned with predefined categories such as “barriers and facilitators”. Simultaneously, the guides were designed to offer flexibility for participants to introduce emergent or unexpected insights, supporting inductive exploration. This dual approach ensured that data collection was both theory-informed and receptive to emerging perspectives.

Data were collected between August and September 2024, after the provision of the psychosocial service. The Principal Investigator (PI), fluent in Amharic and a trained facilitator fluent in both Amharic and Affan Oromo, conducted key informant interviews using tailored guides for each participant group. Interviews included thirteen (13) nurses who delivered PSS, four (4) healthcare extension workers who conducted home visits, and five (5) medical professionals (oncologists, general surgeons, and gynecologists) who were involved in the provision of PSS. Key informant interviews were conducted at healthcare facilities and offices. In-depth interviews were held with seven (7) patients who had received various PSS components, and were conducted either at healthcare facilities or participants’ homes, ensuring privacy and confidentiality. A focus group discussion was also conducted at a healthcare facility. All interviews and discussions lasted 20 to 100 minutes, and were voice-recorded, transcribed, and translated into English.

### Data analysis

Data analysis employed a hybrid thematic approach that combined deductive and inductive approaches, as outlined by Fereday and Muir-Cochrane (11). Analysis was captured through memo writing by the researchers and subsequently discussed within the team. Multiple reviews of transcripts and voice records were conducted to ensure familiarization with the data. All transcripts were imported into NVivo software (version 15) for qualitative data analysis and assistance with data organization. To ensure consistency and reliability in the coding process, two researchers independently coded a subset of the transcripts. Inter-rater reliability was assessed, with iterative rounds of coding and discussion to reconcile discrepancies and refine the coding scheme. Predefined themes, “barriers” and “facilitators”, were introduced deductively based on the research objectives and existing literature, providing a structural framework for organizing the data. Within these overarching categories, inductive coding was used to allow subthemes to emerge naturally from participants’ narratives. This approach enabled the research team to remain grounded in the data while also engaging with theoretical constructs relevant to the study. The decision to adopt a hybrid approach was driven by the need to both validate known challenges in psychosocial service delivery and uncover context-specific insights that may not be captured in existing frameworks. This strategy enhanced the study’s methodological rigor by ensuring both analytical depth and contextual sensitivity (12, 13). The analysis followed the steps of thematic analysis described in Braun and Clark (14) for qualitative data analysis. Data reporting followed COnsolidated criteria for REporting Qualitative research (COREQ) (15).

### Trustworthiness

All interviews were recorded to ensure credibility and enable precise capture of participant responses (16, 17). Probing and clarification techniques were employed to enhance the trustworthiness of the data. An audit trail was maintained through systematic organization of the data and iterative refinement of the codebook (18). Dependability and confirmability were supported by consistent documentation of analytic decisions and efforts to minimize researcher bias (16). Transferability was maintained through purposive sampling, enabling meaningful interpretation across similar settings (17).

### Reflexivity and positionality

During interviews and focus group discussions with cancer patients, the researcher engaged in in-depth conversations about patients’ experiences throughout their cancer treatment journey and daily lives. Due to the researcher’s empathetic nature, it was sometimes challenging to continue conversations when sensitive topics arose and, breaks were sometimes necessary during interviews to manage emotions. To mitigate this, we employed reflexive practices throughout data collection and analysis, including regular debriefing with a research assistant and external experts not involved in data collection. In addition, the researcher holds strong views on women’s rights and strongly opposes the societal expectations placed on women in Ethiopia. For example, when healthcare extension workers explained that they counseled women with cervical cancer to continue to meet their husbands’ sexual needs, despite their chronic condition, it triggered difficult emotions. Hence, the researcher had to be mindful of certain reactions in these situations. With the continuous support of an expert research assistant and experts who were not involved in data collection, we were able to reflect on the researcher’s positions and bring this perspective into the analysis in a scientific manner (19).

### Ethical considerations

Participants provided written informed consent following a detailed explanation of the study’s purpose and procedures. Furthermore, to protect the confidentiality and privacy of the study participants, we pseudonymized characters that might identify the personal traits of individuals. Ethical approval for the present study was obtained from the School of Public Health, Addis Ababa University, and the institutional review board of Addis Ababa University, College of Health Sciences, with protocol number 071/24/SPH.

## Results

A total of 22 participants were involved in the Key Informant Interviews. Most of the participants had an educational background of at least a degree, with over 10 years of work experience (**Table 1**). Among the participants involved in the In-depth Interviews and Focus Group Discussion, the majority were breast cancer patients with an educational background of primary school level or highers (**Table 2**). Barriers and facilitators to the provision of psychosocial service were identified at different levels (**Table 3**).

**Table 1:** Sociodemographic characteristics of participants in the key informant interviews.

Characteristics	Categories	Number of participants (N = 22)
Age	27-31	2
	32-36	14
	>37	6
Sex	Female	12
	Male	10
Educational status	Diploma	1
	Degree	10
	Masters and above	11
Year of experience	3–6 years	7
	7–10 years	6
	>10	9

**Table 2:** Sociodemographic characteristics of participants in the in-depth interviews and focus group discussion.

Characteristics	Categories	Number of participants (N = 14)
Age	30-40	4
	41-50	7
	51-62	3
Sex	Female	11
	Male	3
Educational status	No Formal Education	4
	Primary Level Education	3
	Secondary level Education	4
	Degree	3
Marital status	Single	3
	Married	11
Religion	Orthodox Christian	6
	Muslim	3
	Protestant Christian	5
Occupation	Unemployed	2
	Housewife	4
	Daily Laborer	3
	Pensioner	2
	Government employed	3
Cancer type	Breast Cancer	9
	Cervical Cancer	1
	Colorectal Cancer	4

**Table 3:** A roadmap of themes, subthemes, and codes.

Themes	Subthemes	Codes
Barrier to the provision of psychosocial support	Individual/patient-related	Lack of awareness of psychosocial support
		Patients’ personal circumstances and logistical challenges
	Healthcare provider-related	Cultural Misconceptions
		Resistance to home visits
		Communication gap
		Knowledge gaps in psychosocial support provision
	Institutional	Human resource limitation
		Space constraint
Facilitators to the provision of psychosocial support	Individual/patient-related	Relatability and relevance of audiovisual materials
	Healthcare provider-related	Healthcare professionals’ experience and education
		Training and Supervision
		Provision of travel reimbursements and refreshments

### Barriers to the provision of psychosocial services

#### Lack of awareness about psychosocial support

Because of limited awareness of the importance of psychosocial support, cancer care is often considered to involve only medical treatments, such as chemotherapy and surgery. Consequently, the value of psychosocial support is frequently overlooked. Healthcare professionals reported that they consider medical treatments to be the sole valid approach to cancer care, while many patients were either unaware of available counseling services or believed that they were not essential.

*“Due to a lack of awareness about the importance of counseling, patients are focused on completing their treatment and rushing back home, avoiding the services we provide.”* ***(KII01, Oncology nurse)***

Hospital administrations also prioritize physical treatments such as chemotherapy and radiotherapy for cancer care over psychosocial services. Due to limited awareness of its importance, psychosocial support is frequently undervalued, rarely promoted and seen as an additional task rather than an integral part of cancer care. As a result, support activities such as group discussions are often overlooked or poorly scheduled, frequently taking place during lunch hours or other inconvenient times, placing added strain on staff and disrupting established routines.

“*There is a lack of awareness among hospital management about the importance of psychosocial support. They think psychosocial support provision is an empty promise because they are too busy trying to fulfil the physical treatment needs.” (KII19*, *Oncologist)*

*“The discussions were held at lunchtime, which interfered with our routines. We had to get back to work immediately. We were often told by management to hurry up and finish quickly so that our main work wouldn’t be disrupted.” (KII02, Oncology nurse)*

*“Not everyone knows about the availability of such support in this setting. Several patients might have used the supportive discussions” (FGD, Colorectal cancer patient)*

Professional misunderstandings regarding the purpose and scope of psychosocial support, such as counseling, have led some professionals to perceive it as an administrative duty rather than a meaningful approach to addressing patients’ emotional and psychological needs, which further contributes to its marginalization. As a result, some healthcare providers reduce psychosocial support to casual conversation, overlooking its clinical significance in comprehensive care.

*“We met them because we were conducting a study at the time. We just spoke with them briefly when they visited.” (KII03, Oncology nurse)*

*“There is not much change we can bring by only talking if it is not backed by clinical management and treatment.” (KII16, Gynecologist)*

#### Cultural misconceptions

Cancer is often viewed as a death sentence, shaped by limited awareness and past experiences. As a result, families tend to avoid discussing cancer or the diagnosis. Cultural misconception further discourages open dialogue, especially in semi-urban areas, where norms dictate who may speak about serious illnesses and how.

In such settings, as part of psychosocial support provision, children were sometimes expected to explain cancer-related information to their illiterate parents using brochures, but this proved difficult and was often not done.

*“Despite the brochure being very helpful in explaining what cancer is and the side effects of treatments, in our culture, children often feel afraid to discuss serious matters like cancer with their parents. As a result, children did not read or discuss the informative materials with their parents.” (KII05, BSc Nurse)*

Furthermore, cultural conditioning influences how men and women experience and process emotions, and affects behavior in various settings, including healthcare environments. Healthcare providers observed that women were not only more present but also more engaged during the support group discussions than men. Several factors were suggested to explain this: 1) women tend to be more sociable and open to sharing their experiences; and 2) many women expressed that their voices were not heard at home, making the group discussions a valuable space for emotional expression.

*“Women were active and participated more than the men. They were sociable, laughing, sharing their experiences, and appeared to be happier. The discussion felt as lively and engaging as conversations during coffee ceremonies.” (KII13, MSc nurse)*

Nurses also noted that group discussions became an emotional outlet for women participating in the study, especially those dealing with cervical cancer. They expressed feelings of embarrassment and isolation within their homes due to the symptoms of their illness, such as foul-smelling vaginal discharge, and the discussions provided them with a rare chance to voice these concerns in a supportive environment.

*“Women with cervical cancer told me they felt embarrassed around their husbands due to their symptoms, because of the foul-smelling discharge. At home, they felt like their concerns were unheard, while the men’s issues are acknowledged and often amplified. In contrast, during group discussions, they found relief and communicated openly, expressing their experiences in a supportive environment.” (KII18, BSc nurse)*

#### Resistance to home visits

Home visits play a crucial role in psychosocial support because they address patients’ needs within the comfort of a familiar environment. While patients are generally accustomed to home visits for maternal and child healthcare services, the concept of home visitations remains unfamiliar for chronic conditions such as cancer. This lack of familiarity often leads to confusion about the purpose of the visits and, in some cases, unrealistic expectations such as expecting financial aid.

Furthermore, due to the stigma associated with their illness, and because they do not want their name to be associated with the disease, some patients refused home visits even after completing medical treatment. Despite assurances of confidentiality, they consistently refused to be contacted by health extension workers (HEW) or other health professionals regarding their cancer condition. This reluctance may reflect a lack of trust in the healthcare providers’ ability to safeguard their privacy and maintain discretion.

*“Because psychosocial support is a new concept for many, there was initial resistance towards NCD home visits. Patients often expected financial aid rather than medical support, leading to challenges in establishing the purpose of the visits.” (KII14, Health Extension Worker)*

*“Some patients insisted on avoiding any contact, determined to keep their cancer diagnosis private, even after recovery. They were afraid of breaches of confidentiality and prioritized their privacy above all else.” (KII09, BSc nurse)*

Despite the fact that some patients were reluctant to accept home visits, HEWs do play a pivotal role in delivering this essential service. However, their ability to provide effective home-visit care is frequently hindered by logistical challenges, including long travel distances, inaccurate patient addresses, and limited contact details. Although HEWs reside within the community they serve and possess contextual familiarity, coverage gaps persist. In certain settings, patients live at addresses that fall beyond the urban HEWs’ designated catchment zones, while some rural HEWs had to discontinue their roles due to personal circumstances. Consequently, patients in these areas were left without adequate follow-up care.

*“Locating patients’ houses was difficult. There were occasions where they pointed me in one direction, saying it was nearby, but it turned out to be far away. Even after searching for a few days, I was unable to find the house.” (KII14, Health Extension Worker)*

#### Patients’ personal circumstances and logistical challenges

Patients have expressed that the severity of their disease takes an emotional and physical toll on them, which, along with their social responsibilities and work commitments, often makes it challenging to attend psychosocial support activities, especially group discussion sessions. These factors can make it difficult for them to prioritize participation in these sessions, since they are focused on managing their health while also juggling their daily routines and social responsibilities. Healthcare professionals have also mentioned that patients’ individual circumstances often hinder their participation in group discussion sessions.

*“Personal circumstances, whether due to illness or social obligations like visiting family or attending social gatherings, can make attending support group discussions difficult.” (IDI04, Breast cancer patient)*

*“I have a young child who is handicapped and entirely dependent on me, as I am her sole caregiver, which makes my participation in the discussions difficult!” (FGD, cervical cancer patient)*

*“Some individuals were unable to attend due to their severe illness or because they went to other places for treatment or because they were visiting their children.” (KII09, BSc nurse)*

Healthcare providers have also highlighted that irregular attendance, patients leaving sessions early, and lack of punctuality among patients disrupt the continuity of group discussion sessions, making it challenging to create a cohesive environment. Patients arriving late or missing sessions interrupts the flow of the discussions, reducing their effectiveness and the overall benefits of the support. Healthcare providers also face challenges as these disruptions interfere with their primary duties in busy outpatient departments, creating a conflict between their routine responsibilities and providing psychosocial support.

*“Some patients missed sessions, while late arrivals disrupted group discussion sessions; and, punctuality issues clashed with our work, as we needed to return to other responsibilities” (KII02, Oncology nurse)*

Irregular attendance and lack of punctuality may be due to transportation issues and challenges related to the unique complexities of rural areas, which create significant barriers that often discourage patients from attending psychosocial support sessions.

*“The reason why they don’t come after saying they will is because of the issue of transportation, so if we schedule 10 people, 6 might come.” (KII03, Oncology nurse)*

#### Communication gap

Effective communication is crucial in cancer care, because it facilitates timely decision making and more personalized patient support. Yet, delays in essential services such as radiotherapy, combined with poor communication, often leave patients unaware of alternative treatment options. This can lead to missed chances for medical intervention and disease progression that could have been prevented. Psychosocial support, including counseling about treatment choices, is frequently neglected, further contributing to patient frustration and mistrust in the system.

*“I worried about delaying radiation, but after years of waiting, I considered private care. By then, three doctors told me it was too late, and the treatment wouldn’t help. I wish I had been told earlier! If I were advised about my options, I would’ve started radiation right after chemotherapy and sought private care.” (IDI01, Breast cancer patient)*

Furthermore, the inability of healthcare providers to communicate in patients’ native languages, especially in referral settings, poses a significant challenge. Language barriers often lead to miscommunication, with crucial details lost in translation, leaving patients confused and unsupported throughout their cancer treatment journey. This dissatisfaction affects the quality of psychosocial support, hindering the ability of healthcare providers to address serious issues effectively and empathetically.

*“I am not fluent in Afan Oromo, which hinders me from transferring information and reassuring patients. When I try to talk in Afan Oromo, it seems like I am joking or not serious, which is a huge barrier during counseling and prevents me from transmitting my message.” (KII13, MSc nurse)*

Clear communication is vital to effective psychosocial support in cancer care. However, cultural norms, family pressures, and professional hesitancy around disclosure hinder open dialogue, limiting patients’ ability to cope with their cancer diagnosis and treatment. Despite training in psychosocial communication, many nurses default to routine counseling and avoid disclosing cancer diagnoses, often viewing it as outside of their professional role. This creates a significant communication gap, leaving patients uninformed about their condition, treatment expectations, and potential side effects, even while undergoing care.

*“*First and foremost, it is not advisable for nurses to disclose a diagnosis to the patient, but instead this should be done by the diagnosing physician; because they are the ones diagnosing the disease and have enough knowledge about the patient’s condition.” *(KII02, Oncology nurse)*

Although families play a crucial role in cancer care, their influence also often complicates the cancer diagnosis disclosure process. Caregivers frequently request that healthcare providers withhold diagnoses, believing that patients are too fragile to handle the emotional impact of disclosure. By restricting open discussions, patients are denied essential information about their diagnosis, treatment options, and the emotional support they need. Furthermore, healthcare providers sometimes face institutional directives to respect caregivers’ wishes regarding disclosure. Physicians often justify these decisions as part of “individualized care” for cancer treatment, where the approach to disclosure varies depending on patient and caregiver preferences. While this approach aims to prioritize patient and caregiver comfort, it frequently leads to a lack of transparency that undermines the effectiveness of psychosocial support initiatives.

*“Families insist we hide the diagnosis, fearing the patient can’t handle the news. I often comply, afraid of worsening their condition if I go against their wishes.” (KII02, Oncology nurse)*

*“Some patients complete their treatment without even knowing their diagnosis. We have tried discussing this issue with the physician. We were told that cancer care is individualized care, and it’s not like other diseases. It varies for each individual case, and it can be very challenging. Because of that, if the family requests us not to inform the patient, we comply with that.” (KII01, Oncology nurse)*

#### Knowledge gap in psychosocial support provision

Training in the provision of psychosocial support was generally well-received; however, some healthcare workers felt that training sessions lacked sufficient practical guidance, particularly with respect to counseling techniques. Although training covered a broad range of topics, a common criticism was that it did not offer enough detailed instruction on how to counsel patients.

*“We were trained the most on the theoretical part; we did not have practical sessions with patients while receiving training on counseling.” (KII21, MSc nurse)*

Healthcare providers often felt unequipped to address complex inquiries from patients, particularly health extension workers, who frequently encounter questions that exceed their training, such as survival times, treatment effectiveness, and nutritional advice. Lacking the necessary expertise, they are often left unable to provide the kind of comprehensive answers that patients desperately seek.

*“When I go for a home visit, they ask about their chances of survival … I struggle to respond to that because I don’t have the knowledge to answer such inquiries.” (KII08, Health Extension Worker)*

Nutrition is another critical area where healthcare providers feel unprepared to give advice. Patients frequently seek dietary advice, yet providers struggle to offer clear, evidence-based recommendations.

*“They ask mostly about the recommended diet, which is important. We used to tell them what we know, but they wanted strong confirmation.” (KII03, Oncology Nurse)*

Married women with cancer often face additional challenges related to sexual intimacy. Cervical cancer patients, in particular, report frustrations stemming from their husbands’ lack of understanding and continued expectations for intimacy despite the physical and emotional toll of this illness. Health extension workers frequently encounter such concerns during home visits. In many cases, health extension workers offer advice based on personal beliefs rather than evidence-based, knowledgeable guidance, emphasizing the preservation of marital relationships over patient well-being. Broader societal expectations compound the challenges faced by women with cancer. Regardless of their health status, women are expected to fulfill caregiving and traditional wifely duties. These pressures add significant emotional and physical strain, making it even harder for patients to focus on their recovery. Although health extension workers aim to support patients, they may inadvertently reinforce societal norms by advising women to prioritize their husbands’ needs, often due to limited knowledge or training.

*“Beyond their illness, patients want us to listen about their lives, particularly how their condition affects their husbands. Cervical cancer patients often express frustration that men fail to understand the pain related to that area. They talk about this extensively, sometimes to the point where there is nothing left to say.”* (*KII11*, *Health extension worker)*

*“Mostly, when cervical cancer patients tell me about their intimacy issues with their husbands, I advise them to take care of their husbands because, after all, this is their life. So, I just advise them to suggest that their husbands search for a solution because she can’t provide what he is asking, and for her to accept that, that is how we help.” (KII17, Health extension worker)*

#### Human resource limitations

High workloads and insufficient staffing are critical barriers to the sustainable provision of psychosocial services. As a result, despite receiving training, many healthcare providers stop offering psychosocial services due to their other overwhelming clinical responsibilities. Although healthcare professionals across various facilities received psychosocial support training, in some settings such services were discontinued shortly after training due to competing clinical duties. Healthcare professionals often find themselves juggling psychosocial support duties alongside their regular clinical responsibilities, leaving little time or energy to focus on this critical aspect of patient care. The absence of dedicated resources further compounded the issue, making it difficult to provide consistent and effective psychosocial support.

*“There aren’t many dedicated resources for psychosocial support. Currently, it’s often handled by professionals who juggle it with their other routine responsibilities. Many individuals stopped working after receiving the training because of their other routine clinical responsibilities.” (KII09, BSc nurse)*

In rural areas, the situation was even more pronounced. Health extension workers, who initially received training on how to conduct home visits, were often faced with increased workloads when their colleagues left for personal reasons, such as relocation or childbirth. The added responsibilities forced the remaining health extension workers to schedule home visits beyond regular working hours, to include weekends and late nights.

*“Because the one working with me relocated for personal reasons, I had to work outside my regular schedule, on weekends, or after normal working hours, which makes my schedule very busy.” (KII14, Health extension worker)*

In referral facilities, high patient flow and overwhelming workload presented additional challenges. Healthcare providers in these settings often struggled to find the time needed to offer psychosocial support services. Many healthcare professionals pointed out that the high volume of patients left them with little opportunity to engage with patients on a personal level. As a result, despite their training and good intentions, healthcare professionals were often unable to deliver the psychosocial care that patients needed, further highlighting the significant human resource barriers to effective provision of psychosocial services.

*“Sometimes, patients might not get the chance to see us due to time constraints and routine clinical activity.” (KII04, BSc nurse)*

*“The high patient flow and heavy workload make it difficult to provide psychosocial support, these are our biggest challenges. (KII03, Oncology nurse)*

#### Space constraints

Overcrowding in clinical oncology settings often limits privacy, hindering the delivery of effective psychosocial support. Shared spaces and chaotic environments force healthcare professionals to improvise, disrupting meaningful conversations and leaving both providers and patients feeling unsupported.

Another critical issue is the absence of designated counseling rooms in many facilities. In the absence of private, quiet spaces for sensitive discussions, healthcare professionals often find themselves “begging for rooms” to conduct counseling sessions. This lack of a dedicated area for psychosocial support undermines the quality of care, as counseling takes place in public or shared spaces where confidentiality is compromised. Without adequate space, meaningful, private conversations become increasingly difficult, reducing the overall effectiveness of the psychosocial care provided.

*“All patients are initially checked in a single OPD, where nurses and surgeons work together. Because it’s not practical to talk to them under such conditions, I would make the patients wait until after their consultation, because of which they might have to stay until the evening to get counseling.” (KII05, BSc Nurse)*

“There isn’t a designated area, so we end up talking to them wherever we can, which is challenging. Having meaningful conversations with patients in public areas like the hallway is not motivating.” *(KII02, Oncology nurse)*

### Facilitators to the provision of psychosocial support

#### Training and supervision

Psychosocial support training equips providers with the means to understand and respond to the emotional dimensions of cancer care, ultimately transforming the way healthcare professionals approach patient support.

*“Before the training, I had no experience in handling my patients’ emotions. But afterwards, I learned how to build strong interpersonal relationships with them and better understand the emotional burden each patient carries.” (KII18, BSc nurse)*

Another crucial factor in the successful provision of psychosocial support is close supervision and support of healthcare professionals during supportive supervision. Nurses and staff members emphasized the importance of timely assistance and regular follow-ups from project implementers during support provision. When uncertainties or challenges arose, access to immediate guidance enabled healthcare providers to remain focused and maintain the necessary level of psychosocial support for patients.

*“Consistent supportive follow-ups and sustainable supervision have significantly impacted my commitment and the quality of care I provide.” (KII05, BSc nurse)*

#### Relatability and relevance of audio-visual materials

Patients expressed appreciation for survivor stories that were displayed during group discussion sessions. Audio-visual materials showcasing actual patient survivor stories and experiences are highly relatable, enhancing their appeal and the preference for such formats. Similarly, healthcare providers also noted that patients were excited while watching the survivor stories, which helped to promote more active discussions among the patients.

*“I remember the video vividly … She shared her treatment journey as a cancer patient and how both her breasts were removed … Her powerful message made me see cancer as just another disease, something we shouldn’t let overwhelm us. I used to worry about my situation, but after watching her video, I learned to accept it. There are those in worse conditions, and that realization brought me peace.” (IDI02, Breast cancer patient)*

#### Provision of travel reimbursements and refreshments

Regardless of whether they come from urban or rural areas, cancer patients face significant challenges in terms of time and financial constraints that make it more difficult to attend healthcare appointments. Travelling to health facilities for medical care often disrupts patients’ daily routines, causing stress and imposing additional costs. However, one important facilitator in overcoming these barriers is the provision of transportation reimbursements for patients as well as healthcare professionals.

*“We used to compensate patients for their transportation costs after they attended support group discussions, which eased their attendance in the group discussions.” (KII12, BSc nurse)*

*“My transport expenses were covered when I travelled to them, or theirs will be covered when they come to us.” (KII09, BSC nurse)*

Coffee ceremonies are a deeply rooted tradition in Ethiopian culture; and are regarded as a symbol of respect and hospitality, and a means of fostering meaningful connections. These ceremonies were incorporated into support-group discussions to create a culturally enriched and welcoming environment. Nurses observed that patients responded positively, expressing appreciation for the incorporation of these cultural ceremonies because they evoked a sense of home and belonging, which helped patients feel more at ease discussing emotional issues.

*“What we witnessed during that time was that the tea and coffee ceremony helped us a lot with communication. People love coffee! People love ceremony! So, when you prepare things like that, people come and discussions will be easier.” (KII03, Oncology nurse)*

#### Suggestions for improvement

Healthcare professionals reported that the training program lacked sufficient practical sessions. They suggested extending the training program to include more hands-on learning and requested additional periodic training sessions to ensure comprehensive and up-to-date knowledge.

*“I don’t believe that I received extensive training, as it was for a very brief period. Additional periodic training is necessary.” (KII09, BSc nurse)*

*“Practical training will enable us to have a better experience in counseling and improve our understanding of the patients.” (KII21, MSc nurse)*

Healthcare professionals highlighted the need for psychosocial support training to be extended across all departments involved in cancer care; and emphasized the importance of involving healthcare administration and management and helping them recognize its value and support its integration into practice.

*“Involving managerial staff as well as each department in the training would further strengthen the initiative by addressing system-level issues.” (KII05, BSc nurse)*

In the absence of standardized guidelines, healthcare professionals often rely on personal experience, leading to inconsistent psychosocial support provision. Providers highlighted the need for a clear manual to ensure consistent and effective psychosocial care.

*“Initially, the psychosocial support provision was irregular, but through repetition, we learned the process. A manual would help us improve and stay up to date.” (KII13, MSc nurse)*

## Discussion

This study has identified several barriers to the provision of psychosocial support for cancer patients, including: limited awareness, cultural misconceptions, resistance to home visits, patients’ personal circumstances, logistical challenges, communication problems, and knowledge gaps. Conversely, key facilitators included: targeted psychosocial support training, relatability and relevance of audio-visual materials, distribution of travel reimbursements and refreshments, and supervision.

We found that limited awareness of the availability and significance of psychosocial support services was a key barrier to the effective provision of such support services. This finding is in agreement with previous studies, indicating that low awareness of supportive care within the healthcare setting impedes its utilization and the ability of healthcare professionals to provide psychosocial support services (20, 21). This observed awareness gap may be attributed to inadequate integration of psychosocial services in the healthcare system, and insufficient outreach activities designed to raise patient awareness and understanding regarding the availability and potential benefits of psychosocial support services in cancer care. Misconceptions about psychosocial support have emerged as a key barrier in the this study. Despite its recognized importance, psychosocial support is often given a low priority in cancer treatment settings. Consistent with this, previous research has also indicated that healthcare providers tend to place greater emphasis on medical and routine bedside care, addressing psychosocial needs only when time permits (22). The marginalization of psychosocial support in cancer care may originate from the greater emphasis placed on biomedical treatment modalities, where clinical priorities often focus solely on patients’ physical outcomes. Further, the absence of structured frameworks for psychosocial support integration hinders its systematic inclusion in oncology services, leading to its delivery being relegated to overburdened nurses or staff who often lack specialized training. Consequently, psychosocial care is frequently overlooked.

Previous studies have shown that patients often tend to avoid psychosocial support services due to lack of cultural sensitivity (23, 24). They also emphasized a need for more culturally sensitive cancer support programs, since these can increase patient engagement and service utilization (25). Similarly, in our study, cultural misconceptions and taboos were identified as barriers to the provision of psychosocial support via informational brochures, because some cultures are not accustomed to open communication between patients and their children regarding difficult conditions like cancer. In addition, this study revealed that gender stereotypes shaped by cultural norms influenced participation in supportive care activities, with men less likely than women to engage in support group discussions. This finding is in line with previous studies indicating that women are generally more comfortable expressing emotional concerns, whereas men tend to be more reserved (26, 27). Further, studies have identified that, generally, men are less likely to seek help for health issues than women (28). This also applies to receiving psychological support (29). Although there are various reasons for this, perceptions that men should be strong or that mental illness is less serious than physical illness can be mentioned (30). Such reluctance may arise from cultural notions, which are deeply rooted in communities that associate emotional expression and seeking support as female traits and portray emotional restraint as a marker of masculinity.

In our study, some participants resisted certain components of psychosocial support, such as home visits. This was due to several factors, including the social stigma surrounding their illness, reluctance to have their names associated with cancer, and lack of awareness about the purpose of the home visit. In support of this finding, previous studies on psychosocial service provision for patients and their caregivers indicated that stigma-related fears can represent a significant barrier, as patients may avoid utilizing these services to prevent being recognized as someone with the disease condition (25, 31). This resistance may arise from patients’ lack of trust in healthcare providers, as some do not believe their medical information will be safeguarded, even by professionals. Another reason may be the failure of nurses to inform patients during counseling about the planned follow-up home visits by health extension workers. Patients should be informed first before conducting home visitations, since lack of awareness and consent can lead to resistance.

Clear and effective communication between healthcare providers and patients is essential for cancer treatment. However, our study revealed that psychosocial support, including counseling about the cancer diagnosis, treatment choices and prognosis, often remains neglected, leaving patients uninformed, and resulting in frustration and mistrust in the healthcare system. Similarly, a study in Australia found that lack of communication, including healthcare providers’ reluctance to hold difficult conversations with their patients, is a major barrier to providing effective psychosocial care (32). This reluctance to share critical information with patients may result from healthcare providers’ desire to avoid the emotional burden associated with confronting issues beyond their control, such as treatment unavailability, prognosis, which falls outside of the scope of psychosocial services. Further, nurses also identified language as a barrier to the provision of psychosocial support that impedes effective communication. Similarly, previous studies have shown that when healthcare professionals do not speak the patients’ native language, significant communication challenges arise, hindering the delivery of quality psychosocial care (22, 33).

Family involvement in diagnosis disclosure was identified as a barrier to providing effective counseling services for patients. Caregivers often request that healthcare providers withhold diagnoses, because they believe that patients are too fragile to handle the emotional impact. As a result, patients remained uninformed about their condition and miss opportunities to receive psychosocial support from healthcare providers. Similarly, studies conducted in the Middle East have shown that frequent ‘do not tell’ requests from families influence the patient disclosure process, often resulting in physicians disclosing the diagnosis to family members rather than directly to the patient (34, 35). Another study focusing on family requests for nondisclosure, which focused on the case of low- and middle-income countries, indicated that families often request nondisclosure to protect patients from emotional distress, especially in cases of serious illness (36). Further, a scoping review of clinical communication in cancer care among 19 African countries reflects that cultural orientations toward communalism over individualism, which is rooted in the Ubuntu philosophy of interconnectedness, which is common across diverse African settings, indicates that open discussion about diagnosis, prognosis and end-of-life or death is taboo in many places (37). As a result, families interfere with the disclosure process, which is a common challenge in these settings (38). Moreover, a study conducted in Ethiopia on the preferences of patients, families, and the general public regarding cancer diagnosis disclosure revealed that while patients wish to be informed about their diagnosis and poor prognosis and prefer that oncologists do not withhold such information, family caregivers often prefer that this information be withheld from patients (39). Caregiver influence may stem from societal norms that support withholding serious illness-related information or bad news from affected individuals. While not universal, these norms are often rooted in a desire to shield the person from emotional distress, fear, or potential social stigma.

In this study, nurses reported feeling unprepared to respond to patients’ inquiries during psychosocial support sessions, largely due to insufficient training and limited knowledge. Similarly, a previous study indicated that healthcare providers’ ability to provide supportive care services is significantly influenced by the professionals having proper knowledge to engage in such activities (40). In addition, another study on the potential barriers to psychosocial care provision revealed that nurses’ feelings of inadequacy was another barrier that prevents the provision of psychosocial support care (22).

Moreover, heavy workloads due human resource shortages and high staff turnover were also identified as barriers to the effective provision of psychosocial support in the present study. In line with our findings, studies examining the educational needs of inpatient oncology nurses providing psychosocial care also identified heavy workloads and staff turnover as primary barriers, leaving nurses with limited time to offer psychosocial support (41–44), because busy clinical schedules often limit the ability of clinicians to engage in meaningful conversations with patients.

The lack of designated space for counseling was also reported by each of the healthcare facilities included in the present study as a critical barrier to the provision of psychosocial services. In support of this, a previous study also concluded that the lack of private space for counseling was a significant barrier (44), because confidential settings are necessary to maintain patient privacy during counseling sessions.

Our findings revealed that various factors contribute to the effective provision of psychosocial support activities. Key facilitators identified in the current study included implementation of training programs on how to provide psychosocial support, as well as the relatability and relevance of psychosocial support materials. Resources that depict real patient experiences tend to be more engaging and preferred by service users. Similarly, previous studies have shown that interventions tailored to the specific needs of the target group are widely appreciated and properly utilized by participants (45, 46). Other key facilitators the provision of psychosocial support identified in the current study include travel reimbursements for attendees, the provision of refreshments, and regular supervision for professionals. These findings are supported in the literature, where financial incentives to participants attending sessions (25), the availability of refreshments (47), and supervision (44) have all been identified as major facilitators that support the provision of psychosocial support activities.

A key strength of this study is the inclusion of both cancer patients and healthcare professionals, providing a comprehensive perspective on the barriers and facilitators of psychosocial service delivery. Furthermore, the study incorporates diverse data collection methods, verbatim transcription, and iterative review of data, which strengthen the credibility of our results. However, a notable limitation is the potential for translation bias. Transcribing and translating interviews from Amharic and Afan Oromo into English may have resulted in the loss of nuanced meanings, which could affect the depth and accuracy of our understanding of the perspectives and cultural expressions of study participants.

## Conclusions and recommendations

This study identified limited awareness of psychosocial services as a key barrier to their effective provision and utilization. This finding highlights the importance of targeted outreach efforts in increasing awareness among patients and healthcare staff about the availability and benefits of cancer support services. Moreover, cultural misconceptions and taboos were found to impede supportive care, emphasizing the need to develop culturally sensitive educational materials to promote acceptance and engagement. Gender-related differences were also identified as influential factors, with stereotypical norms affecting patients’ willingness to engage in emotional communication. Supportive care programs should therefore adopt gender-sensitive approaches to enhance patient-clinician communication by recognizing these differences. Furthermore, patient resistance to home visits was linked to a lack of prior information about the visits and fear of stigma. This underscores the necessity for clear communication between healthcare providers and patients regarding the purpose and nature of the home visits. Building trust is also crucial to reassure patients that their confidentiality will be maintained. Lastly, communication gaps, especially concerning the disclosure of diagnosis, treatment options, and prognosis, were identified as barriers. This highlights the need for enhanced training in clinician–patient communication, both in professional practice and in the academic curricula. In addition, structured approaches, such as private conversations with family members, may help address the concerns of family members and support ethically sound disclosure practices within cancer care settings. Because the lack of private, designated spaces in oncology settings significantly impedes the delivery of confidential and effective psychosocial support; facilities should prioritize the establishment of dedicated counseling rooms to enhance the quality of PSS while maintaining patient dignity.

## Data Availability

The raw data supporting the conclusions of this article will be made available by the authors, without undue reservation.
